# Physical Functioning, Depressive Symptoms, and Suicidal Ideation among Older Korean Adults

**DOI:** 10.3390/ijerph18168781

**Published:** 2021-08-20

**Authors:** Hyung-Seop Sim, Sang-Gyu Lee, Tae-Hyun Kim

**Affiliations:** 1 Department of Public Health, College of Medicine, Yonsei University, Seoul 03722, Korea; shs221194@naver.com; 2 Department of Preventive Medicine, College of Medicine, Yonsei University, Seoul 03722, Korea; leevan@yuhs.ac; 3 Department of Healthcare Management, Graduate School of Public Health, Yonsei University, Seoul 03722, Korea

**Keywords:** physical function, activities of daily living, instrumental activities of daily living, depressive symptom, suicidal ideation

## Abstract

Previous studies have shown that the physical functioning of older adults directly affects their depressive symptoms, and suicide is also closely associated with depression. This study determined the effects of physical functioning on depressive symptoms and suicidal ideation among older Korean adults. This study used data from the 2017 National Survey of Older Persons. Among the 10,299 participants in the entire data set, 10,083 participants were analyzed, excluding 216 participants who did not respond to the dependent variables. Data analyses included frequency, chi-squared tests, and binary logistic regression. The results indicated that physical functioning among older adults was associated with reduced depressive symptoms and suicidal ideation. Compared to the group that had non-limited activities of daily living (ADL) function, the group with limitations was 1.66 times more likely to show depressive symptoms (OR: 1.66, 95% CI: 1.36–2.02). Similar trends were observed in instrumental activities of daily living (IADL) (OR: 1.85, 95% CI: 1.58–2.16). When suicidal ideation was set as a dependent variable, IADL had a statistically significant impact (OR: 1.41, 95% CI: 1.14–1.74); however, ADL did not seem to have an impact. Moreover, both ADL (OR: 1.62, 95% CI: 1.35–1.94) and IADL (OR: 1.72, 95% CI: 1.49–1.97) had statistically significant effects when combined with depressive symptoms and suicidal ideation. Better physical functioning was associated with a reduction in depressive symptoms and suicidal ideation. This study emphasizes the importance of physical functioning when examining older adults’ mental health.

## 1. Introduction

Depression is a major health risk factor for older adults [1], among whom the rates of depression and frailty are high [2]. This is not a unique trend in Korea. Korea’s aging rate is expected to reach 43.9% by 2060, and the level of aging is expected to be very high [3]. Korea is already an aging society, with 15.7% of the population aged 65 years and above in 2020, and the proportion of the aging population continues to rise [4].

In Korea, the suicide rate has increased for those aged 70 years and above compared to other age groups [5]. Furthermore, suicide is closely associated with depression [6]. According to a meta-analytic study on suicide, the prevalence of mental disorders among suicide cases was 80.8% [6]. Additionally, in the National Survey of Older Persons (NSOP) 2014, 33.1% of senior citizens aged 65 years or above had depressive symptoms, and 10.9% reported that they had considered suicide. Moreover, 12.5% of the older adults who had suicidal thoughts had attempted suicide [7].

An increase in the population of older adults implies an increase in the population wherein individuals’ sensory, cognitive, and physical functions decrease [8,9]. Previous studies have shown that physical functioning tends to decrease with age [8,9]. In the NSOP 2014, 43.7% of the survey respondents aged 65 years or above believed that their health was poor [7]. In fact, the number of older adults with some limitations in the performance of activities of daily living (ADL) or instrumental activities of daily living (IADL) accounted for 18.2% of the total respondents [7].

As the number of elderly people increases, the number of individuals complaining of depression also increases [10]. Previous studies have shown that the physical limitations of older adults directly affect their experience of depressive symptoms [11,12,13], and that there is a significant correlation between the two variables [11,14,15]. 

Additionally, there is a significant difference in the level of depression depending on the ADL and IADL of older adults [11], with some studies showing that the number of chronic diseases and physical functioning of older adults are important factors in predicting depression [14]. A study conducted on older adults with sarcopenia showed that those with lower body functioning had significantly higher depression scores [12]. Another study, which compared a group of older adults with disabilities and considerable limitations in physical functioning, observed a 1.72-fold higher prevalence of depression than those without disabilities [13]. A previous study highlighted that physical function alone did not explain depression; however, there was a significant correlation between the two [15]. 

Results from the NSOP 2014 showed that a significant proportion of older adults had depressive symptoms and suicidal ideation [7]. There is a significant association between depressive symptoms and suicidal ideation, and people with depressive symptoms may experience suicidal ideation [6]. However, few studies have examined how the physical functioning of older adults affects both depressive symptoms and suicidal ideation. Additionally, most studies conducted in Korea collect data through surveys, and the samples used are not representative of the entire population of older adults of the country. For example, older adults residing in certain facilities have not been surveyed to the same extent as their counterparts have [11,12,14].

The current study determined the effects of physical functioning on depressive symptoms and suicidal ideation among older adults in Korea using the NSOP 2017. In particular, this study determined how the limited physical functions of the older adults affect depressive symptoms and suicidal ideation.

## 2. Materials and Methods

### 2.1. Description of the Sample

This study used raw data from the NSOP 2017 conducted by the Korea Institute for Health and Social Affairs (KIHASA), which conducts a survey every three years according to the Senior Welfare and Welfare Act. It aimed to provide basic data necessary for policymaking for older adults.

The survey was stratified based on the place of residence of all older adults in the country; thus, it was representative. Additionally, data were systematically collected by trained researchers participating in various national surveys.

The survey participants were older adults aged over 65 years living in general residential facilities nationwide. The survey was conducted between 12 June 2017 and 28 August 2017.

The number of survey districts and older adults who completed the survey was 879 and 10,299, respectively. Of these, the responses of 10,083 participants were used for the analysis, because the remaining 216 were missing data (surrogate respondents) in depressive symptom variables.

### 2.2. Measures

The dependent variables were depressive symptoms and suicidal ideation. The Korean version of the Short Form of the Geriatric Depression Scale (SGDS-K) was used to measure depressive symptoms among older adults. The sum of 15 items (yes = 1, no = 0; total score range 0–15) about whether participants had felt depressed in the past week indicated depressive symptoms. A score of 8 or higher was defined as the presence of depressive symptoms. Suicidal ideation consisted of a response to the question asking if the older adult had ever thought of suicide since the age of 60.

The key independent variables were physical functions of older adults, defined as ADL and IADL. The ADL were measured using seven items: dressing, washing face, bathing and showering, eating food, lying down, getting off the toilet, and controlling their bowels. The sum of the seven items’ scores was defined as follows: those with 7 points were classified as the “Non-limited” group, and those who scored 8 or more points were classified as the “Limited” group. 

The IADL were measured using 10 items: grooming, housework, meal preparation, laundry, taking medication, managing money, community mobility, purchasing goods, making and receiving calls, and using transportation. The sum of the 10 items’ scores was defined as follows: those who scored 10 points were classified as the “Non-limited” group, while those with 11 or more points were classified as the “Limited” group.

In most studies, ADL and IADL have been used as continuous variables [9,13,14,16,17]. However, some studies have used ADL or IADL categorized by scores [11,12,18]. In particular, in Shin’s study, older adults who did not need help at all were classified as the “normal-range group”, and those who responded that they needed help in one or more items were classified as the “impaired group” [18]. In this study, we attempted to determine the effects of limited physical functioning on depressive symptoms and suicidal ideation among older adults, referring to Shin’s classification of ADL and IADL.

The control variables were socioeconomic and health behavioral factors. Socioeconomic factors included sex, age, spouse, education level, employment status, and the number of chronic diseases. “Spouse” indicated whether or not a spouse was present, and “Education level” was divided into uneducated, elementary school graduation, middle school graduation, and high school graduation or higher. “Employment” indicated whether the subject was currently working or not. The number of chronic diseases was defined as the total number of chronic diseases diagnosed by a doctor.

Health behavior factors included smoking, drinking, and exercise habits. Smoking indicated whether an individual currently smoked. Drinking alcohol indicated whether an individual currently drank alcohol at the same frequency as the previous year.

### 2.3. Statistical Analysis

Data were analyzed using the Statistical Analysis System (SAS) 9.4 (SAS Institute Inc., Cary, NC, USA). The frequency and percentage of respondents according to their socioeconomic factors, health behavior factors, and physical functions were calculated. Statistical differences between each variable were confirmed using the Pearson’s chi-squared test. Binary logistic regression analysis was performed to confirm the effects of physical functioning on depressive symptoms and suicidal ideation among older adults.

## 3. Results

### 3.1. Depressive Symptoms and Suicidal Ideation of the Study Population

Table 1 displays the general characteristics of the 10,083 survey participants included in this study. Of the surveyed participants, 40.1% (n = 4046) were men and 59.9% (n = 6037) were women. A total of 10.5% (n = 426) of men and 12.6% (n = 759) of women had experienced depressive symptoms, and the proportion of women was higher than that of men (*p* = 0.002). The proportion of women with suicidal ideation was approximately 1% higher than that of men (*p* = 0.014).

Among the participants, those who were older, did not have a spouse, had a lower education level, and were currently unemployed tended to have a higher proportion of depressive symptoms (all significant at *p* less than 0.001). A greater proportion of those with chronic diseases had depressive symptoms compared to the reference group (*p* < 0.001). In suicidal ideation, all variables except age showed similar patterns to depressive symptoms, and age showed no statistically significant relationship (*p* = 0.173). Participants who did not have a spouse (*p* < 0.001), had a lower education level (*p* = 0.002), were currently unemployed (*p* < 0.001), and had more chronic diseases (*p* < 0.001) tended to have a higher proportion of suicidal ideation. 

Regarding health behavior factors, those who did not consume alcoholic beverages in the past year tended to be more depressed than those who consumed alcohol (*p* < 0.001). The group that did not exercise regularly tended to be more depressed (*p* = 0.001). There were no statistically significant differences concerning smoking (*p* = 0.449). 

In terms of physical functioning, the higher the limitation for both ADL (*p* < 0.001) and IADL (*p* < 0.001), the higher the proportion of older adults with depressive symptoms (Table 1).

Regarding health behavior factors, those who smoked were more likely to think about suicide than those who did not (*p* = 0.016). The group that did not exercise regularly tended to think more about suicide (*p* = 0.05). There were no statistically significant differences concerning drinking (*p* = 0.663). In terms of physical functioning, the higher the limitation for both ADL (*p* < 0.001) and IADL (*p* < 0.001), the higher the proportion of suicidal ideation among older adults.

### 3.2. The Effect of Physical Function on Depressive Symptoms and Suicidal Ideation

Table 2 presents the results of the binary logistic regression model analyzing the effects of physical functioning on depressive symptoms and suicidal ideation. 

Regarding ADL, the limited group was 1.66 times more likely to have depressive symptoms than the non-limited group (OR: 1.66, 95% CI: 1.36–2.02). Concerning the IADL, the limited group was 1.85 times more likely to have depressive symptoms than the non-limited group (OR: 1.85, 95% CI: 1.58–2.16). The physical functioning of older adults affected their depressive symptoms and suicidal ideation. In IADL, the limited group was 1.41 times more likely to think of suicide than the non-limited group (OR: 1.41, 95% CI: 1.14–1.74). ADL had no statistically significant effect on suicidal ideation (OR: 1.26, 95% CI: 0.94–1.68).

However, by combining depressive symptoms and suicidal ideation into one variable, the results were different. ADL (OR: 1.62, 95% CI: 1.35–1.94) and IADL (OR: 1.72, 95% CI: 1.49–1.97) were found to have a statistically significant effect on the combined variables.

In the present study, the group without a spouse was more depressed than the group with a spouse (OR: 1.52, 95% CI: 1.31–1.76), and the higher the education level, the lower the rate of feeling depressed. Additionally, the group without a spouse was more likely to think about suicide than the group with a spouse (OR: 2.06, 95% CI: 1.70–2.48). The unemployed group had a higher level of depressive symptoms and suicidal ideation than the employed group. Those with a higher number of chronic diseases were more likely to have depressive symptoms and suicidal ideation. Concerning exercise, the group that did not exercise regularly showed a higher level of depressive symptoms than the group that exercised regularly.

## 4. Discussion

This study attempted to determine how the physical functioning of older adults affects their depressive symptoms and suicidal ideation using data from the NSOP 2017. 

Due to the analysis, in both ADL and IADL, the ‘Limited’ group was more likely than the ‘Non-limited’ group to have depressive symptoms. The results of this study are consistent with those of previous studies [11,12,14,16,18]. The physical functioning of older adults, as represented by ADL and IADL, was associated with depressive symptoms. 

Regardless of the presence or absence of depressive symptoms, physical activity is essential for daily life. People with physical problems report that their daily lives become harder when they have depression [11]. Furthermore, those with disabilities were 72% more depressed than those without disabilities [13]. This seems to be due to limitations in physical functioning.

Many studies suggest that the majority of those with physical illness have additional psychiatric disorders [2,11,12,13,14,19]. A previous study found that the risk of suicide increases if someone has a mental illness along with a physical illness [19]. The results of the present study showed that older adults with limited physical functioning had a higher rate of depressive symptoms and suicidal ideation. This result is consistent with previous studies which show that suicidal ideation and behaviors increase if someone has a particular physical condition [19,20].

Concerning the health of older adults, the management of their physical functioning is vital for their mental health [21,22]. It is natural for bodily functions to decline with age. However, this decline in physical functioning can be prevented by physical activity [9,22,23]. Exercise has an effective antidepressant action and has a positive effect on mental health [24].

A decline in physical function is also linked to a decline in physical activity [12]. To prevent this decline, a standard physical activity program for older adults in Korea is being promulgated to senior citizens to promote physical activity and create a health-friendly environment for them [3]. However, this is only conducted for older adults who visit the welfare centers. Older adults whose physical functioning is too limited to visit the welfare center are in the blind spot of the program. To prevent this, mental health risk groups should be selected for older adults based on their ADL and IADL.

At the community level, there is a need for healthcare programs for at-risk older adults that restore their physical functioning through regular and continuous exercise activities. It is worth noting that socioeconomic factors are significantly related to depressive symptoms and suicidal ideation among older adults. People with a low socioeconomic status are more likely to have mental illness, have less access to health care, and less satisfaction with their overall health status [18,21,25]. This trend is not unique to Korea. The impact of SES increases with age. This is called the “Matthew Effect”, [26], which differs not only in material resources but also in physical and mental health according to socioeconomic factors. In the present study, the lower education level group had a higher proportion of depressive symptoms and suicidal ideation.

This study has several limitations. Firstly, this was a cross-sectional study, which can explain the correlations between variables to a point, but cannot explain the causality. For example, it is not known whether the limitations in physical functioning affect depressive symptoms and suicidal ideation or whether depressive symptoms and suicidal ideation affect physical functioning limitations. Secondly, although scores of eight or higher were interpreted as being depressed on the SGDS-K [7], it cannot be stated that older adults with a score of six or seven were not depressed. It is important to consider both severe and mild depressive symptoms.

Nevertheless, because this study was based on nationally conducted surveys, the reliability of the survey results is high. The survey was conducted at the national level, not in a specific region; hence, it is representative of the population. Additionally, unlike previous studies, the strength of this study is that it investigated how physical functioning factors affect depressive symptoms and suicidal ideation among older adults.

## 5. Conclusions

More than 20% of Korean older adults experience depressive symptoms, whereas 6.7% experience suicidal ideation. If this number increases with the increasing trend in aging, the management of depressive symptoms and suicidal ideation will become crucial.

The results of this study found that the worse the physical functioning, the higher the level of depressive symptoms and suicidal ideation among older adults. For older adults, early intervention will help them improve their physical functioning and lead to a healthy life.

Currently, Korea is conducting a senior citizen’s exercise support project to prevent deterioration of their physical functioning. Considering the effects of limited physical functioning on depressive symptoms and suicidal ideation, it is thought that targeting and providing customized interventions for the vulnerable is required as a strategy for intervention programs to enhance the physical functioning of older adults.

Older adults with depressive symptoms or suicidal ideation can also be classified as a vulnerable group. When implementing an exercise support project for older adults, it may be considered to provide mental care and physical activities by classifying vulnerable groups.

According to previous studies, the physical functioning of older adults can be supplemented through physical activity. Physical functioning is also related to mental health. Therefore, considering that this age group is at risk of developing mental health issues, attempts are needed to manage their physical functioning.

## Figures and Tables

**Table 1:** General characteristics of the study population (N = 10,083).

Variables	Total		Depressive Symptom				*p*-Value	Suicidal Ideation				*p*-Value
			Yes		No			Yes		No		
	N	(%)	N	(%)	N	(%)		N	(%)	N	(%)	
Sex												
Male	4046	40.1	426	10.5	3620	89.5	0.002	223	5.5	3823	94.5	0.014
Female	6037	59.9	759	12.6	5278	87.4		407	6.7	5630	93.3	
Age												
65–69	2628	26.1	229	8.7	2399	91.3	<0.001	185	7.0	2443	93.0	0.173
70–74	2674	26.5	242	9.1	2432	91.0		166	6.2	2508	93.8	
75–79	2582	25.6	344	13.3	2238	86.7		158	6.1	2424	93.9	
≥80	2199	21.8	370	16.8	1829	83.2		121	5.5	2078	94.5	
Spouse												
No	3786	37.5	604	16.0	3182	84.1	<0.001	335	8.9	3451	91.2	<0.001
Yes	6297	62.5	581	9.2	5716	91.0		295	4.7	6002	95.3	
Education												
Uneducated	2699	26.8	487	18.0	2212	82.0	<0.001	208	7.7	2491	92.3	0.002
Elementary school	3538	35.1	389	11.0	3149	89.0		212	6.0	3326	94.0	
Middle school	1602	15.9	146	9.1	1456	90.9		92	5.7	1510	94.3	
≥High school	2244	22.3	163	7.3	2081	92.7		118	5.3	2126	94.7	
Employment												
No	6880	68.2	978	14.2	5902	85.8	<0.001	478	7.0	6402	93.0	<0.001
Yes	3203	31.8	207	6.5	2996	93.5		152	4.8	3051	95.3	
Number of chronic diseases												
None	966	9.6	37	3.8	929	96.2	<0.001	25	2.6	941	97.4	<0.001
1–2	3791	37.6	324	8.6	3467	91.5		147	3.9	3644	96.1	
≥3	5326	52.8	824	15.5	4502	84.5		458	8.6	4868	91.4	
Smoking												
No	9133	90.6	1081	11.8	8052	88.2	0.449	553	6.1	8580	94.0	0.016
Yes	950	9.4	104	11.0	846	89.1		77	8.1	873	91.9	
Drinking												
No	7569	75.1	955	12.6	6614	87.4	<0.001	478	6.3	7091	93.7	0.663
Yes	2514	24.9	230	9.2	2284	90.9		152	6.1	2362	94.0	
Regular exercise												
No	3346	33.2	553	16.5	2793	83.5	<0.001	232	6.9	3114	93.1	0.050
Yes	6737	66.8	632	9.4	6105	90.6		398	5.9	6339	94.1	
Activities of daily living (ADL)												
Non-limited	9349	92.7	966	10.3	8383	89.7	<0.001	556	6.0	8793	94.1	<0.001
Limited	734	7.3	219	29.8	515	70.2		74	10.1	660	89.9	
Instrumental activities of daily living (IADL)												
Non-limited	7475	74.1	604	8.1	6871	91.9	<0.001	401	5.4	7074	94.6	<0.001
Limited	2608	25.9	581	22.3	2027	77.7		229	8.8	2379	91.2	

**Table 2:** Factors associated with depressive symptom and suicidal ideation.

Variables	Depressive Symptom		Suicidal Ideation		Depressive Symptom × Suicidal Ideation	
	OR	95% CI	OR	95% CI	OR	95% CI
Sex						
Male	1.70	1.44–2.02	1.30	1.04–1.62	1.56	1.34–1.80
Female	Ref		Ref		Ref	
Age						
65–69	1.26	1.02–1.54	2.55	1.95–3.35	1.53	1.28–1.83
70–74	1.01	0.84–1.23	1.86	1.43–2.42	1.20	1.01–1.42
75–79	1.18	0.99–1.40	1.50	1.13–1.93	1.22	1.04–1.43
≥80	Ref		Ref		Ref	
Spouse						
No	1.52	1.31–1.76	2.06	1.70–2.48	1.67	1.47–1.91
Yes	Ref		Ref		Ref	
Education						
Uneducated	1.87	1.51–2.33	1.09	0.83–1.43	1.59	1.32–1.92
Elementary school	1.47	1.20–1.80	1.02	0.80–1.30	1.33	1.12–1.57
Middle school	1.28	1.00–1.62	1.01	0.76–1.35	1.17	0.96–1.44
≥High school	Ref		Ref		Ref	
Employment						
No	1.94	1.65–2.30	1.36	1.12–1.66	1.68	1.46–1.93
Yes	Ref		Ref		Ref	
Number of chronic diseases						
None	Ref		Ref		Ref	
1–2	1.98	1.39–2.82	1.59	1.03–2.45	1.76	1.32–2.35
≥3	3.00	2.12–4.24	3.47	2.28–5.27	3.06	2.31–4.05
Smoking						
No	1.02	0.80–1.28	0.71	0.54–0.92	0.89	0.73–1.08
Yes	Ref		Ref		Ref	
Drinking						
No	1.11	0.94–1.32	0.95	0.77–1.17	1.04	0.90–1.20
Yes	Ref		Ref		Ref	
Exercise						
No	1.53	1.34–1.74	1.09	0.92–1.31	1.39	1.24–1.57
Yes	Ref		Ref		Ref	
Activities of daily living (ADL)						
Non-limited	Ref		Ref		Ref	
Limited	1.66	1.36–2.02	1.26	0.94–1.68	1.62	1.35–1.94
Instrumental activities of daily living (IADL)						
Non-limited	Ref		Ref		Ref	
Limited	1.85	1.58–2.16	1.41	1.14–1.74	1.72	1.49–1.97

Note: OR, odds ratio; CI, confidence interval; Ref, reference group.

## Data Availability

Data are available at http://data.kihasa.re.kr/ (accessed on 3 July 2019).
